# Turkish school teachers’ knowledge and attitudes toward HIV/AIDS

**DOI:** 10.3325/cmj.2012.53.271

**Published:** 2012-06

**Authors:** Naim Nur

**Affiliations:** Department of Public Health, Faculty of Medicine, Cumhuriyet University, Sivas, Turkey

## Abstract

**Aim:**

To assess Turkish school teachers’ knowledge, attitudes, and misconceptions of HIV/AIDS.

**Methods:**

This cross-sectional descriptive study was conducted in Sivas, Middle Anatolian province of Turkey, from January to May 2009. We selected and interviewed 898 teachers using a simple systematic sampling method.

**Results:**

All respondents heard about AIDS. Most knew that it could be transmitted by infected blood (98.0%) and sexual contact (93.4%) and some (33.2%) thought that it could be transmitted by mosquitoes. Although the majority of them strongly agreed or agreed with the statement that “people with AIDS should be helped, supported and treated” (98.0%), about 40% and 70% of them, respectively, agreed or strongly agreed with the statement that infected people should be quarantined. Young teachers with a higher level of knowledge about HIV/AIDS had more positive attitudes.

**Conclusion:**

This study provided basic information on school teachers’ AIDS knowledge and attitudes and showed that various misconceptions of HIV transmission were a matter of concern. A considerable number of respondents with undesirable attitudes toward HIV/AIDS indicates the need for education of teachers on the subject.

Today, human immunodeficiency virus/acquired immune deficiency syndrome (HIV/AIDS) is one of the biggest public health problems, with 31-36 million estimated cases and 2.7 million new cases in 2010 (1). In Turkey, AIDS was first identified in two cases in 1985 and the number of AIDS cases has been increasing ever since. At the end of 2011, there were 4303 HIV-positive individuals in Turkey and 921 AIDS cases (2). However, these data might underestimate the real number, which is likely to be four times higher because of the recording system of illnesses and the fact that HIV has a long asymptomatic period (3). Since transmission through injecting drugs use is rare in Turkey, heterosexual intercourse is the principal route of transmission, followed by blood transfusion (2).

Attitudes toward HIV/AIDS are influenced by various aspects of culture (3). For example, in Turkish society sexual behavior is still considered a taboo subject and having a sexually transmitted disease is considered a shame. However, as a result of social and cultural changes in Turkey, people in some parts of the country exercise greater sexual freedom than previous generations, which puts them at a greater risk of sexually transmitted infections, including HIV/AIDS (4,5).

Health education and prevention of HIV/AIDS remain the main health care priorities in Turkey (5). It can be supposed that adequate knowledge about AIDS would reduce the risk behavior (5,6). However, quantitative studies carried out in Turkey suggested that the level of knowledge about HIV/AIDS was very low (7,8).

Health behavior adopted in childhood appears to have a strong influence on future health activities (9). As more than one third of the population in Turkey is adolescent, their education is the key for prevention programs. Consequently, good education of school children depends on school teachers' views and knowledge about HIV/AIDS. Knowledge creates the precondition for a change of habits. Health behavior theories have added to our understanding of how cognitive and social factors contribute to human health and disease. If people lack awareness of how their habits affect their health, they have little reason to change the bad lifestyle habits they enjoy (10). Teachers have very important role in modifying school children’s knowledge and attitudes toward HIV/AIDS (11).

While several studies on HIV/AIDS-related knowledge, attitudes, and misconceptions have been conducted among university students (2), street children/youths (12), and soldiers (13), there is no data about Turkish school teachers. This study aimed a) to assess school teachers’ knowledge, attitudes, and misconceptions of HIV/AIDS and b) to provide information useful to health workers and policymakers for establishing prevention programs.

## Methods

This cross-sectional descriptive study was conducted in Sivas, Turkey, from January to May 2009. Sivas is a semi rural province situated in the Middle Anatolia, with a population of about 700 000. The city has an average socio-economic level, with considerable disparities between different city quarters (14).

We used a simple systematic sampling method. The government schools were randomly selected from the alphabetical list of schools at the Department of Education of Sivas. The first school was randomly selected from the list, followed by every subsequent fifth school (ie, 1st, 6th, 11th, etc). From 178 middle and high schools, we systematically selected 38 (with 937 teachers). All teachers working in these schools were contacted and invited to participate. Permission to conduct the research was obtained from the headmasters. Teachers were explained that their participation in the study would be completely voluntary, that it would not affect their employment, and that all information would be confidential. A total of 937 teachers were approached and 39 refused to participate. The response rate was 95.8%. The information for the main study was collected anonymously using self-administered questionnaires, which were distributed to each school by eight trained final-year medical students. The study was approved by the Human Research Ethics Committee of the Cumhuriyet University of Turkey.

The questionnaire was developed based on a literature review, reviewed by a specialist in infectious diseases and a general physician, and pre-tested in one school. Cronbach alpha coefficient for the reliability (internal consistency) of the subscales (as used in this study) ranged from 0.72 to 0.83 (15). The questionnaire consisted of 3 sections: demographic data and previous sources of knowledge about HIV/AIDS and willingness to learn (7 items), disease knowledge including mode of transmission, misconceptions, and methods of prevention and control (23 items), and attitudes toward HIV/AIDS patients (14 items). The response options were “yes,” “no,” and “do not know” (one point was given for a correct answer and zero for an incorrect or no answer). Each answer was assigned a score of 0 or 1. The total possible cumulative score ranged from 0 to 23. The cumulative score was re-coded into dichotomous variables (<18 = insufficient and 18 or over = sufficient) based on the mean value of 18.2 ± 2.3. For assessing attitudes, a 5-point Likert scale was used (5 = strongly agree to 1 = strongly disagree) (16).

Data were analyzed using SPSS (SPSS Inc., Chicago, IL, USA) for Windows, version 13.0. Quantitative data were presented as mean ± standard deviation. Categorical data were expressed as percentages. χ^2^ tests were performed to determine the differences between demographic items (age groups, sex, marital status, and teaching experience) and knowledge levels or attitudes. A linear regression model was also performed to assess the influence of predictor variables that had been significant in univariate analysis on the knowledge level and attitudes toward HIV/AIDS. All statistical tests were conducted at the 5% significance level.

## Results

### Respondents’ characteristics

All respondents lived in the urban area and had university degrees (Table 1). Of 898 respondents, 448 (49.9%) were female and 670 (74.6%) were married. The mean age of the respondents was 36.5 ± 8.1 years, ranging from 19 to 60 years and 499 (55.6%) had a 10-year or longer teaching experience. All had heard about HIV/AIDS, but more than a half thought that they needed more knowledge. The main sources of information were television, newspapers, and friends, followed by the internet and health professionals. Respondents believed that their levels of knowledge were sufficient.

**Table 1:** Characteristics of teachers from the city of Sivas included in the study (n = 898)

Characteristics	No. (%)
Age groups (years):	
<35	381 (42.4)
35-44	350 (39.0)
>44	167 (18.6)
Sex:	
female	448 (49.9)
male	450 (50.1)
Marital status:	
married	670 (74.6)
single	189 (21.0)
separated	19 (2.1)
widowed	20 (2.2)
Teaching experience (year):	
≤10	399 (44.4)
<10	499 (55.6)
Teacher’s need of information:	
yes	547 (60.9)
no	351 (39.1)
Source of information:*	
television	596 (66.4)
newspapers	476 (53.0)
friends	405 (45.1)
internet	250 (27.8)
medical professionals	214 (23.8)
books	209 (23.3)
magazines	180 (20.0)
school	79 (8.8)
family	24 (2.7)
Teacher’s level of information (self assessed):	
sufficient	606 (67.5)
insufficient	292 (32.5)

*More than one answer allowed.

### Knowledge and attitudes toward HIV/AIDS

Respondents had a fairly good knowledge about HIV/AIDS on most items. Most knew that HIV was transmitted by sharing needles or a razor (n = 880, 98.0%), blood transfusion (n = 869, 96.8%), sexual intercourse (n = 839, 93.4%), and from an infected mother to the fetus (n = 822, 91.5%) (Table 2). However, only a few knew that it could be transmitted from infected mother via breast milk (n = 232, 25.8%).

**Table 2:** Participant’s knowledge on HIV/AIDS (n = 898)

	No. (%) of respondents who answered the question
General knowledge*	
AIDS is a preventable disease	824 (91.8)
AIDS is a fatal disease	818 (91.1)
Correct use of condoms is an effective way to prevent the transmission of AIDS during sex	804 (89.5)
The risk of transmission can be reduced by having sex with only one faithful, uninfected partner	797 (88.8)
AIDS is a infectious disease	794 (88.4)
Resistance to other diseases in an individual with AIDS is rather low	784 (87.3)
Person with AIDS live ten years or more after diagnosis	647 (72.0)
A person infected with HIV does not usually show any symptoms of the disease	632 (70.4)
AIDS is an immune system disease	607 (67.6)
AIDS is a genetic disease	60 (6.7)
**Mode of transmission**	
Having sexual intercourse with an infected person	839 (93.4)
Sharing injection needles or razor with an infected person	880 (98.0)
Receiving blood from an infected person	869 (96.8)
From an infected mother to her fetus during pregnancy	822 (91.5)
From infected mother to baby via breast milk	232 (25.8)
**Misconception about transmission**	
The bite of a mosquito	298 (33.2)
Shaking hands or touching an infected person	246 (27.4)
Sharing a meal with someone who is infected	199 (22.2)
Sharing same toilets with an infected person	188 (20.9)
Going into swimming pools used by an infected person	154 (17.1)
Through coughing or sneezing of an infected person	98 (10.9)
**Treatment**	
There is a treatment for AIDS	207 (23.1)
There is a vaccine for AIDS	113 (12.6)
**Best method for prevention and control of AIDS**	
Education and condom use	552 (61.5)
Education and diagnosis together	165 (18.4)
Diagnosis by screening	69 (7.7)
Cure	45 (5.0)
Don’t know	117 (13.0)

*Respondents who agreed with the statement.

A considerable number of the respondents were not aware that HIV infection was not transmitted by mosquito bites (n = 298, 33.2%), shaking or touching hands (n = 246, 27.4%), sharing meals (n = 199, 22.2%), using the same toilets (n = 188, 20.9%), going into same swimming pools (n = 154, 17.1%), and coughing or sneezing (n = 98, 10.9%). A total of 552 (61.5%) respondents believed that the best method for preventing HIV infection was education and condom use.

There were no sex differences in the knowledge on AIDS, except that men responded significantly better on the following items: “AIDS is a preventable disease” (93.7% vs 89.8%, *P* = 0.044) or “there is a treatment for AIDS” (20.2% vs 25.9%, *P* = 0.042). There was no significant difference between other characteristics (age groups, marital status, and teaching experience) and the knowledge level (data not shown).

Respondents had relatively negative attitudes toward people with AIDS (Table 3). The majority strongly agreed or agreed only with the statement that “people with AIDS should be helped, supported and treated” (98.0%). However, a significantly low proportion of respondents agreed with the statements, such as: “people with AIDS should be quarantined” (39.8%) or “students with AIDS should go to special schools for those with AIDS” (70.0%). Additionally, there was no significant difference between demographic characteristics (age groups, sex, and marital status) and attitudes (data not shown). However, linear regression analysis showed that the age of teachers was independently associated with their knowledge score and attitudes. Young teachers with a higher knowledge score about HIV/AIDS had more positive attitudes (Table 4 and Table 5).

**Table 3:** Respondents ’attitudes toward HIV/AIDS (n = 898)

	No. (%) of respondents who		
Statements	strongly agreed/ agreed	strongly disagreed/ disagreed	neither agreed nor disagreed
Students with AIDS should go to special schools for those with AIDS	628 (70.0)	174 (19.4)*	96 (10.6)
If there is a student with AIDS in a school, I would remove my child from that school	559 (62.2)	218 (24.3)*	121 (13.5)
I would not sit side by side with a person with AIDS	360 (40.1)	230 (25.6)*	308 (34.3)
I would not kiss someone with AIDS	626 (79.7)	112 (12.5)*	160 (17.8)
Patients with AIDS should be quarantined	348 (39.8)	289 (32.2)*	251 (28.0)
I would not play a game with a person with AIDS	357 (39.7)	284 (31.7)*	257 (28.6)
I would have personal contact with someone with AIDS as an ordinary person	387 (43.1)*	291 (32.4)	220 (24.5)
I would share public toilets and swimming pools with someone with AIDS	134 (15.0)*	428 (47.6)	336 (37.4)
I would wash my clothes with those of an person with AIDS	121 (13.5)*	467 (52.0)	310 (34.5)
I would be uncomfortable if our close neighbor had AIDS	609 (67.8)	147 (16.4)*	142 (15.8)
I would be uncomfortable if my sister wanted to marry a person with AIDS	815 (90.8)	35 (3.9) *	48 (5.3)
I would be uncomfortable if we had a doorkeeper with AIDS	697 (77.7)	101 (11.2)*	100 (11.1)
I would be uncomfortable if my hairdresser or barber had AIDS	794 (88.4)	35 (3.9) *	69 (7.7)
People with AIDS should be helped, supported and treated	880 (98.0)*	10 (1.1)	8 (0.9)

*Positive attitudes.

**Table 4:** Relation between socio-demographic factors and the level of knowledge about AIDS in multiple linear regression analysis

Independent variables	β	*P*	95% confidence interval
Age*	-0.19	<0.001	-0.005 to -17
Sex^†^	0.05	0.12	-0.013 to 0.109
Marital status	0.02	0.56	-0.055 to 0.097

*In years.

†Male = 1, female = 2.

**Table 5:** Relation between socio-demographic factors and attitudes about AIDS in multiple linear regression analysis

Independent variables	β	*P*		95% confidence interval
Age*	-0.13		0.02	-0.034 to -0.003
Sex^†^	0.03		0.33	-0.078 to 0.230
Marital status	0.01		0.82	-0.169 to 0.214

*In years.

†Male = 1, female = 2.

## Discussion

In this survey, most respondents answered correctly on the questions about HIV/AIDS transmission routes, prevention methods, and treatment. However, 11.6% believed that AIDS was not an infectious disease, that it was transmitted by mosquito bites, and that there was a treatment for AIDS. Furthermore, 27.4% indicated that AIDS can be transmitted by shaking hands, sharing meals, or touching an infected person. Other studies also showed very poor general knowledge on HIV transmission among teachers (17) and that only 16.2% of teachers had clear knowledge on HIV transmission and prevention (18). Similar results were also found in other studies, both from Turkey (19,20) and other countries such as Iran, Nepal, and Tunisia (21-23).

The most interesting finding from this study was the fact that the majority of teachers agreed with the statement that “people with AIDS should be helped, supported and treated.” On the other hand, from 40% to 90% of teachers, regardless of sex and age, showed a negative attitude towards people with HIV/AIDS. For example, 40% of teachers believed that people with AIDS should be quarantined. Similar results were found Israel, where 39% of teachers thought that people with AIDS should be kept away from school (24). Also, in France 22% of respondents agreed that people with AIDS should be locked up or isolated in a special center (24). Furthermore, in an earlier population-based study from Iran (25), more than a tenth of the respondents agreed that people with AIDS should not have the right to study or work. The present study showed that 43% of teachers would discontinue having any relation with someone with AIDS. One prior report from Turkey (26) showed that nearly one-third of students declared that they would avoid sitting near HIV-infected people. Moreover, in the present study, many of the surveyed teachers felt discomfort about having an HIV-positive neighbor, doorkeeper, hairdresser, or barber. In this and other studies conducted in Turkey, Israel, and Iran (20,25,27), multivariate regression analysis showed that young teachers with a higher level of knowledge about HIV/AIDS had more positive attitudes.

Data suggest that respondents’ knowledge about HIV/AIDS mostly does not originate from school but from mass media and communication with friends, health professionals, and family members. Other studies on teachers reported similar sources of knowledge (18,21,22). This all indicates that most of the teachers need more information on AIDS.

Because HIV/AIDS-related issues are a sensitive topic in our country; the questionnaire did not include questions related to teachers' risk behaviors or sexual activities, which might have affected the results. This study provides basic information on AIDS knowledge and attitudes among school teachers and shows that various misconceptions of HIV transmission are a matter of concern. The levels of undesirable attitudes about HIV/AIDS indicate a need for changing the Turkish teachers’ attitudes towards persons with HIV/AIDS through education programs organized by the Ministry of Education in accordance with the social and cultural structures of the society.
